# Acute effects of whey protein on energy intake, appetite and gastric emptying in younger and older, obese men

**DOI:** 10.1038/s41387-020-00139-8

**Published:** 2020-10-02

**Authors:** Avneet Oberoi, Caroline Giezenaar, Caroline Jensen, Kylie Lange, Trygve Hausken, Karen L. Jones, Michael Horowitz, Ian Chapman, Stijn Soenen

**Affiliations:** 1 Adelaide Medical School and Centre of Research Excellence in Translating Nutritional Science to Good Health, The University of Adelaide, Adelaide, Royal Adelaide Hospital, South-Australia, SA Australia; 2 Riddet Institute, Massey University, Palmerston North, New Zealand; 3 Centre for Nutrition, Department of Clinical Medicine, University of Bergen, Bergen, Norway; 4 Department of Medicine, Haukeland University Hospital, Bergen, Norway; 5 Faculty of Health Sciences and Medicine, Bond University, Gold Coast, Queensland, Australia

**Keywords:** Randomized controlled trials, Ageing

## Abstract

**Background:**

Obesity is becoming more prevalent in older people. A management strategy in obese, young adults is to increase dietary protein relative to other macronutrients. It is not clear if this is effective in obese, older individuals. Obesity may be associated with diminished sensitivity to nutrients. We have reported that a 30-g whey protein drink slows gastric emptying more, and suppresses energy intake less, in older, than younger, non-obese men. The aim of this study was to determine the effect of a 30 g whey protein drink on energy intake, GE and glycaemia in obese, older and younger men.

**Methods:**

In randomized, double-blind order, 10 younger (age: 27 ± 2 years; BMI: 36 ± 2 kg/m²), and 10 older (72 ± 1 years; 33 ± 1 kg/m²), obese men were studied twice. After an overnight fast, subjects ingested a test drink containing 30 g whey protein (120 kcal) or control (2 kcal). Postprandial gastric emptying (antral area, 2D Ultrasound) and blood glucose concentrations were measured for 180 min. At *t* = 180 min subjects were given a buffet meal and *ad libitum* energy intake was assessed.

**Results:**

Older subjects ate non-significantly less (~20%) that the younger subjects (effect of age, *P* = 0.16). Whey protein had no effect on subsequent energy intake (kcal) compared to control in either the younger (decrease 3 ± 8%) or older (decrease 2 ± 8%) obese men (age effect *P* > 0.05, protein effect *P* = 0.46, age × protein interaction effect *P* = 0.84). Whey protein slowed gastric emptying, to a similar degree in both age groups (50% emptying time: control vs. protein young men: 255 ± 5 min vs. 40 ± 7 min; older men: 16 ± 5 min vs. 50 ± 8 min; protein effect *P* = 0.001, age effect *P* = 0.93, age × protein interaction effect *P* = 0.13).

**Conclusions:**

Our data suggest that obesity may blunt/abolish the age-related effect of whey protein on suppression of energy intake.

## Introduction

While the number of older people is increasing worldwide^1,2^, it is not as well appreciated that obesity rates are rising across the age range. Approximately one-third of people over 65 years are obese (body mass index (BMI) > 30 kg/m^2^) in Australia^3^ and other developed countries (US, UK etc)^4,5^ and this proportion is increasing. Although the BMI range associated with lowest morbidity and mortality increases with age^6^, a BMI higher than 30 kg/m^2^ in older people is still associated with adverse health outcomes, including diabetes, hypertension and heart disease. Obesity is therefore a serious problem in older people^7–10^ and intentional weight loss is often recommended for obese, older adults^11^.

Ageing is also associated with changes in feeding behaviour, gut function, and body composition, which impact on life expectancy and quality^12–17^. Skeletal muscle mass decreases, while fat mass increases^18^. Insufficient protein intake in obese older individuals exacerbates muscle loss^19^. This muscle loss, which may lead to sarcopaenia, is associated with functional impairment, increased rates of falls, increased nursing home admissions, and other adverse outcomes^10,20–26^.

Protein supplements, often rich in whey protein, are commonly part of weight-loss strategies, based on the rationale that protein is more satiating than the other macronutrients^27–30^, although that effect may be modest, particularly when the protein is consumed as part of typical, ad libitum eating situations. Whey protein is high in essential amino acids which are rapidly digested, resulting in postprandial amino acid availability, stimulating muscle protein accretion more effectively than casein and casein hydrolysate in older men^31,32^. We have recently shown that the acute administration of 30 g (120 kcal) and 70 g (280 kcal) oral whey-protein loads suppressed subsequent energy intake by 12–17% in young people without suppression in healthy older men^33^. The whey-induced suppression of appetite and energy intake in young people may reflect an increase in pyloric and reduction in antral and duodenal motility, factors important in the regulation of gastric emptying^34,35^. Obesity appears to have minor, inconsistent, effects on gastric emptying^36–38^, whereas gastric emptying is modestly slower in older, than younger, adults^33^. The potential influence of gastric emptying and energy/ protein load of an ingested meal on subsequent voluntary food intake is complex and likely to depend on the time of subsequent eating, the degree of gastric distension and initiation of small intestinal mechanisms to stimulate satiety after food leaves the stomach^37,39,40^.

Information about the effects of whey protein on energy intake in obese, older people is limited. We have reported that the acute suppression of energy intake by whey protein, administered either intraduodenally^17^, or orally^33^, is less in healthy, non-obese, older than younger men, suggesting that the use of protein supplements to promote weight loss may not be as affective in older as younger adults^33^. However, ad libitum energy intake responses to oral whey protein have not been evaluated in older, obese, adults. These may have implications for the use of high protein dietary strategies to manage obesity and maintain muscle mass in older people.

The primary aim of this study was to determine the effects of a 30 g whey protein drink on energy intake in obese older and younger men.

## Materials and methods

Ten younger [Mean ± standard error of mean (SEM): age: 27 ± 2 years; body weight: 112 ± 8 kg; height: 1.75 ± 2.81 m; BMI: 36 ± 2 kg/m^2^] and ten older (age: 72 ± 1 years; body weight: 103 ± 4 kg; height: 1.76 ± 2.56 m; BMI: 33 ± 1 kg/m^2^) obese men were recruited by advertisement. Body weight and BMI of the younger and older subjects did not differ significantly (*P* > 0.05).

On the basis of our previous study in lean subjects^17^, we determined that ten subjects/group would be sufficient to detect a difference in the suppression of energy intake by 30 g whey protein of 395 kcal, with standard deviations (SDs) of 316 kcal (younger subjects) and 180 kcal (older subjects)^17^, and in 50% gastric emptying time (T50 min) of 80 min with (SD’s) of 38 min (younger subjects) and 63 min (older subjects)^41–43^, between younger and older subjects, with α = 0.05 and power of 80%.

Subjects were excluded on the basis of smoking, alcohol abuse, diabetes (HbA1C > 6%), significant gastrointestinal surgery, gastrointestinal symptoms (pain, reflux, diarrhoea, or constipation), and the use of medications known to affect energy intake, appetite, or gastrointestinal motor function. For older people, additional exclusion criteria were impaired cognitive function (score < 25 on Mini Mental State^44^) and depression (score ≥ 11 on the Geriatric Depression Questionnaire^45^).

The Royal Adelaide Hospital Human Research Ethics Committee approved the protocol which was conducted in accordance with the Declaration of Helsinki. The study was registered with the Australian New Zealand Clinical Trial Registry (www.anzctr.org.au, registration number ANZCTR12616001216404). All subjects provided written informed consent prior to their study inclusion.

### Protocol

Subjects were studied twice, separated by ≥3 days, to determine the effects of a whey protein drink (30 g/120 kcal) and a control drink (∼0 g whey protein/∼2 kcal) on energy intake, in randomized order (1 block with balanced permutations; www.randomization.com), double-blind, and cross-over design.

The protein drink (∼450 mL) was prepared by dissolving whey protein (Bulk Nutrients, Tasmania, Australia) in demineralized water and diet lime cordial (Bickford’s Australia, South Australia (SA), Australia) to achieve the desired load [i.e. 30 g whey (volume of the powder: 19 mL) in 335 mL distilled water and 85 mL cordial (2.5 kcal/100 mL). The ‘control’ drink contained 0 g protein, 360 mL water, and 90 mL cordial. Sodium chloride, 0.3 g and 1.2 g, was added to the whey and control drinks so that the osmolarity (88 mOsm/L) was matched. To ensure even mixing drinks were stirred continuously at low speed on a stirring plate. The volumes of the drinks differed slightly (control: 450 mL; 30 g protein: 439 mL). Drinks were prepared by a research officer not involved in data analysis and served in a covered cup, so that both investigators and subjects were blinded to the treatment.

Subjects were provided with a standardized meal [beef lasagne (McCain Foods Pty Ltd, Wendouree, Victoria (VIC), Australia), ∼591 kcal] to consume on the night before each study day at ∼1900h. They were instructed to fast overnight ~12 h from solids and liquids except water, and to refrain from strenuous physical activity until they attended the laboratory at ∼0830 h.

On arrival, subjects were seated in a chair, where they remained throughout the study day, and an intravenous cannula was inserted. Measurements of antral area and perceptions of appetite and gastrointestinal symptoms were performed immediately before (during fasting; 0 min), immediately after ingestion of the drink (5 min), and then at 15 min intervals until 180 min. Subjects consumed the drink within 2 min. Antral area was measured by 2-dimensional (2D) ultrasonography^33^. Perceptions of appetite and gastrointestinal symptoms were assessed using visual analogue scales (VAS) and blood samples were collected to measure blood glucose. At 180 min, subjects were given a cold, buffet-style meal, as described^33^, in excess of what they were expected to consume (total energy content of 2,457 kcal; 19% protein, 50% carbohydrates, 31% fat) and instructed to eat freely for up to 30 min until comfortably full (180–210 min)^33^.

### Measurements

#### Energy intake

The amount eaten (g) was quantified by weighing the meal before and after consumption. Energy intake (kcal) and proportions of intake of protein, carbohydrate, and fat were calculated using commercially available software (Foodworks; 3.01, Xyris Software, Highgate Hill, Queensland, Australia). Energy intake was calculated both as intake at the buffet meal and as the cumulative energy intake (sum of energy intake at the buffet meal and from the preload drink). Absolute (kcal) and percentage suppression/change (energy intake as % of control day intake) of energy intake at the buffet meal by 30 g protein compared with control were calculated^16^.

#### Gastric emptying

Gastric emptying was measured at baseline, after overnight fasting (*t* = 0 min), immediately after drink consumption and then every 15 min to 180 min. Gastric emptying rates were calculated from ultrasound measurements of gastric antral area (cm^2^), as previously described^40,46^. Measurements were performed with a Logiq^TM^ ultrasound machine (GE Healthcare Technologies, Sydney, New South Wales, Australia) using a 3.5 C broad spectrum 2.5–4 MHz convex linear array transducer. The transducer was positioned vertically to obtain a para-sagittal image of the antrum with the superior mesenteric vein and the abdominal aorta in a longitudinal section. Measurements were performed at the end of inspiration. To calculate meal retention in the stomach, fasting antral area (measured at baseline) was subtracted from subsequent measurements performed after ingestion of the drinks^46^. Gastric retention was calculated as

$${\mathrm{Retention}}\,(\% ) = [{\mathrm{AA}}({\mathrm{t}}) - {\mathrm{AA}}({\mathrm{f}})]/[]{\mathrm{AA}}({\mathrm{max}}) - {\mathrm{AA}}({\mathrm{f}})^ \ast 100$$

where AA(t) = antral area measured at a given time, AA(f) = fasting antral area, and AA(max) = maximum antral area recorded after drink ingestion^46^. When ultrasound images lacked sufficient clarity, data were imputed by linear interpolation. The time at which 50% of the preload drink had emptied from the stomach (time to halving of post-drink maximum antral area: T50; min) was calculated for both conditions.

#### Perceptions of appetite and gastrointestinal symptoms

Perceptions of hunger, desire to eat, prospective consumption, fullness, nausea, and bloating were rated using a 100 mm visual analogue scale (VAS) questionnaire at *t* = 0, 5, 15, 30, 45, 60, 75, 90, 105, 120, 135, 150, 165, 180, and 210 min as previously described^47^. Area under the curve (AUC) was calculated for appetite ratings (0–180 min) using the trapezoidal rule.

#### Blood glucose

Blood glucose concentrations (mmol/L) were determined by the glucose oxidase method using a portable glucometer (Optium Xceed, Abbott Laboratories, Australia). Area under the curve (AUC) was calculated for blood glucose 0–180 min using the trapezoidal rule.

#### Data and statistical analysis

Statistical analyses were performed using SPSS software (version 25; IBM, Armonk, NY, USA). Effects of age and treatment and their interaction effect were determined using a repeated measures mixed-effects model. An unstructured covariance structure was used to account for the repeated treatments by subject. Suppression of energy intake by protein and outcomes of the control condition were compared between age groups with a paired *t*-test. Statistical significance was accepted at *P* < 0.05. All data are presented as mean values ± SEM.

## Results

The study protocol was well tolerated by all subjects.

### Energy intake

**Fig. 1:**
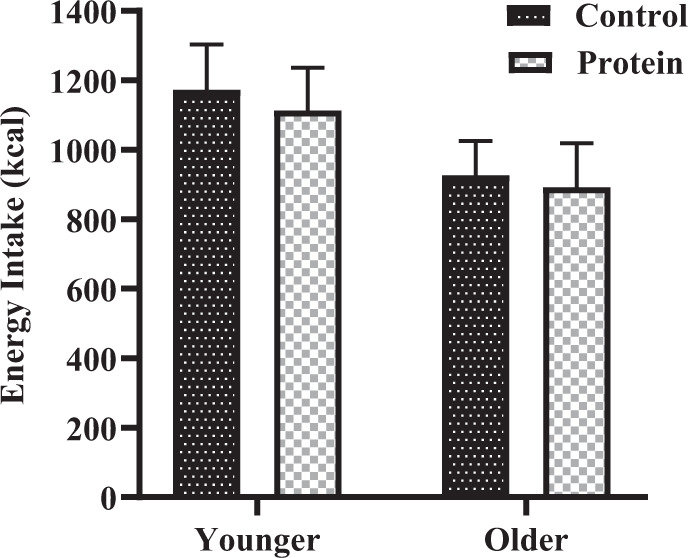
Mean ± standard error of mean (SEM) energy intake at the buffet meal (kcal) in younger (*n* = 10) and older (*n* = 10) men after drinks containing water (control-black) and 30 g whey protein (black border). Main age and protein effects and age × protein interaction effect were determined by using mixed model analysis. The protein drink did not suppress subsequent energy intake at the buffet meal compared with control. The main effects of age (*P* = 0.16) and protein (*P* = 0.46) and the age × protein interaction effect (*P* = 0.84) for energy intake were not significant.

Older subjects consumed ~20% less energy after the drinks than younger subjects, although this difference was not statistically significant (*P* = 0.16). There was no difference in the amount of energy consumed after the protein drink compared to control, in either younger (control 1173 ± 130 kcal vs. protein 1114 ± 124 kcal) or older (control 926 ± 99 kcal vs. protein 892 ± 127 kcal) men (age effect *P* = 0.16, protein effect *P* = 0.46, age × protein interaction effect *P* = 0.84; Fig. 1).

There was no suppression of energy intake by whey protein in either age group (younger −3.0 ± 7.7% vs. older −2.3 ± 8.4%, *P* = 0.95).

Furthermore, cumulative energy intake (intake at buffet meal plus test drink) during the protein day did not differ with age or treatment (age effect *P* = 0.16, protein effect *P* = 0.25, age × protein interaction effect *P* = 0.84).

### Gastric emptying

In one older subject the quality of antral images was insufficient to determine gastric emptying on both days, so data for this subject were excluded from analysis. There was no difference in baseline (after overnight fasting) antral areas between age groups or study treatments (young men control vs. protein: 3.20 ± 0.37cm^2^ vs. 2.98 ± 0.31cm^2^; older men control vs. protein: 2.68 ± 0.29cm^2^ vs. 3.19 ± 0.29 cm^2^; age effect *P* = 0.64, protein effect *P* = 0.61, age × protein interaction effect *P* = 0.23).

**Fig. 2:**
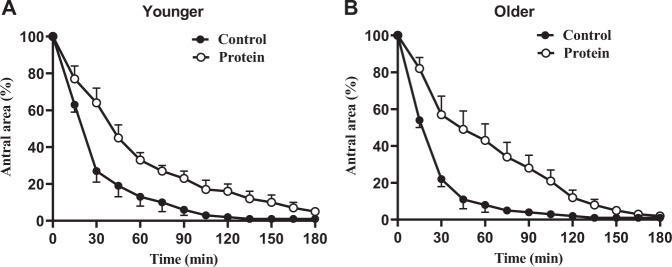
Mean ± standard error of mean (SEM) antral area (%) in younger (*n* = 10) and older (*n* = 10) men after drinks containing water (control) and 30 g whey protein. Main age and protein effects and age x protein interaction effect were determined by using mixed effects analysis. Gastric emptying (antral area) of the protein drink was slower than control to a similar degree in both the age groups. The main effect of protein for 50% gastric emptying time (T50 min; *P* = 0.001) was significant; the age (*P* = 0.93) and the age × protein interaction (*P* = 0.13) effects were not significant.

The protein drink emptied more slowly than control in both groups (T50 young men control vs. protein: 25 ± 5 min vs. 40 ± 7 min; older men control vs. protein: 16 ± 5 min vs. 50 ± 8 min; protein effect *P* = 0.001, age effect *P* = 0.93, age × protein interaction effect *P* = 0.13; Fig. 2). Gastric emptying of the control drink was not significantly different between both age groups (*P* = 0.21).

### Blood glucose concentrations

**Fig. 3:**
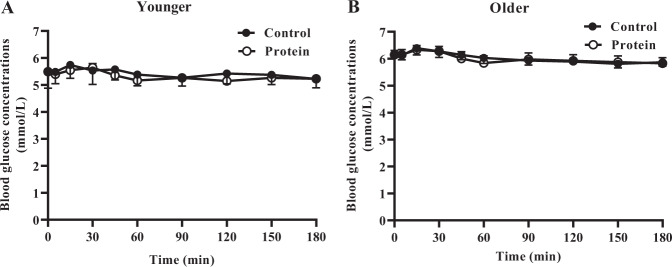
Mean ± standard error of mean (SEM) blood glucose concentrations (mmol/L) in younger (*n* = 10) and older (*n* = 10) men after drinks containing water (control) and 30 g whey protein. Main age and protein effects and age × protein interaction effect were determined by using mixed effects analysis. There was no effect of protein on the blood glucose concentrations in either age group. The main effect of age (*P* = 0.005) was significant; the protein (*P* = 0.42) and age × protein interaction (*P* = 0.54) effects were not significant.

Fasting blood glucose concentrations were higher in older (control 6.1 ± 0.2 mmol/L, protein 6.2 ± 0.2 mmol/L) than younger (control 5.4 ± 0.1 mmol/L, protein 5.4 ± 0.2 mmol/L) men (age effect *P* = 0.003, protein effect *P* = 0.76, age × protein interaction effect *P* = 0.58) and throughout both study days (AUC 0–180 min, young men control: 1003 ± 17 mmol/L, protein: 981 ± 21 mmol/L; older men control: 1112 ± 28 mmol/L, protein: 1108 ± 37, age effect *P* = 0.005). There was no effect of protein on blood glucose concentrations in either age group (protein effect *P* = 0.42, age × protein interaction effect *P* = 0.54; Fig. 3).

### Perceptions of appetite and gastrointestinal symptoms

#### Baseline

Baseline ratings of hunger [younger men control (YC) 53 ± 9 mm, younger men protein (YP) 39 ± 9 mm; older men control (OC): 37 ± 9 mm, older men protein (OP) 44 ± 6 mm], fullness (YC 19 ± 5 mm, YP 20 ± 6 mm; OC 16 ± 6 mm, OP 9 ± 5 mm), nausea (YC 14 ± 6 mm, YP 8 ± 4 mm; OC 4 ± 1 mm, OP 5 ± 1 mm) and bloating (YC 8 ± 3 mm, YP 9 ± 5 mm; OC 7 ± 4 mm, OP 5 ± 1.9 mm) were not different between younger and older men or between control and protein days (all main effects *P* > 0.05).

There were significant but modest age × protein interaction effects for ratings of prospective consumption and desire to eat at baseline. Pairwise comparisons for prospective food consumption (age × protein interaction effect *P* = 0.001) showed that in the young, scores were higher on the control day than protein day, whereas in the older group, scores were higher on the protein day than control day (YC 62 ± 8 mm, YP 51 ± 8 mm, *P* = 0.040 vs. OC 41 ± 8 mm, OP 58 ± 8 mm, *P* = 0.004).

For desire to eat (age × protein interaction effect *P* = 0.020), there was no difference between treatments in either age group (*P* > 0.05). Scores were higher in the younger than the older group on the control day (*P* = 0.040), but not on the protein day (YC 52 ± 8 mm, YP 39 ± 7 mm vs. OC 30 ± 7 mm, OP 39 ± 7 mm, *P* = 0.98).

#### After study drink

##### Prospective food consumption

The main age and protein effects for ratings of desire to eat (total AUC; YC 10,019 ± 1174 mm.min, YP 8,925 ± 1156 mm.min vs. OC 6,003 ± 1173 mm.min, OP 6,763 ± 1156 mm.min; age effect *P* = 0.07, protein effect *P* = 0.71), hunger (YC 10,041 ± 1180 mm.min, YP 8,676 ± 1289 mm.min vs. OC 5,873 ± 1180 mm.min, OP 6,324 ± 1289 mm.min; age effect *P* = 0.07, protein effect *P* = 0.35), fullness (YC 4,705 ± 897 mm.min, YP 4,703 ± 959 mm.min vs. OC 4,884 ± 897 mm.min, OP 4,703 ± 959 mm.min; age effect *P* = 1.0, protein effect *P* = 0.57), prospective food consumption (YC 10,279 ± 1198 mm.min, YP 9,572 ± 1143 mm.min vs. OC 7,341 ± 1198 mm.min, OP 7,607 ± 1143 mm.min; age effect *P* = *0.15*, protein effect *P* = 0.57), bloating (YC 3,151 ± 875 mm.min, YP 2,666 ± 653 mm.min vs. OC 1,973 ± 875 mm.min, OP 1,495 ± 653 mm.min; age effect *P* = 0.28, protein effect *P* = 0.11) were not significant. In younger subjects nausea ratings were higher after the test drink than older subjects (YC 3,127 ± 741 mm.min, YP 2,892 ± 827 mm.min vs. OC 771 ± 741 mm.min, OP 708 ± 827 mm.min; age effect *P* = 0.04). The effect of protein was not significant (*P* = 0.74).

**Fig. 4:**
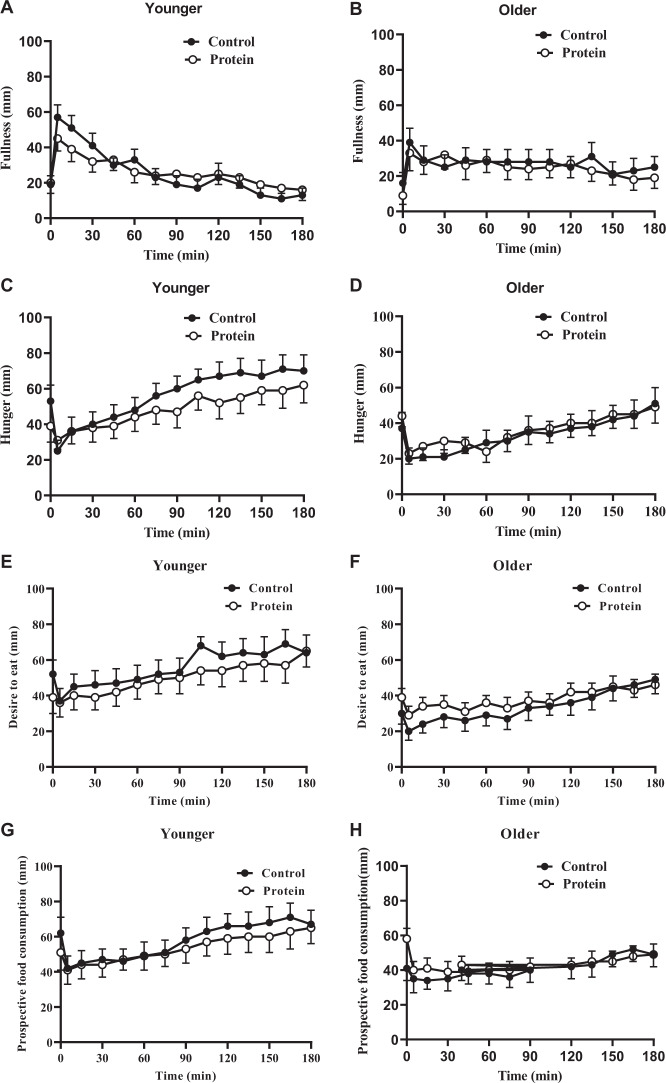

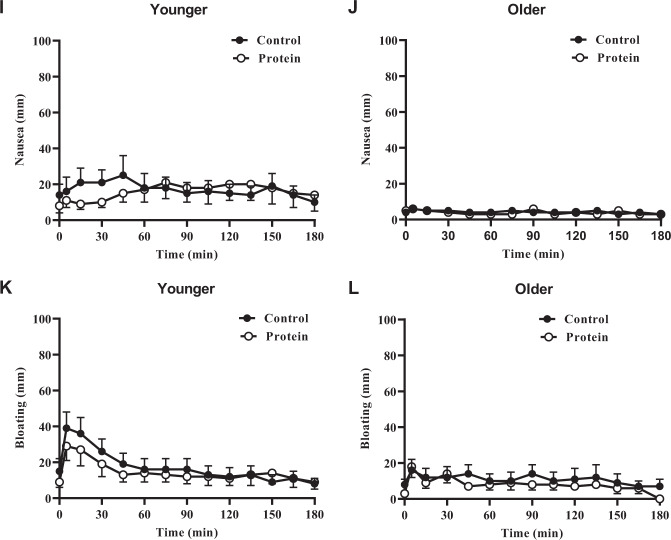
Perceptions of appetite and gastrointestinal symptoms. Mean±standard error of mean (SEM) visual analogue scores (VAS; mm; 0–180min) of fullness (**a**, **b**), hunger (**c**, **d**), desire to eat (**e**, **f**), prospective food consumption (**g**, **h**), nausea (**I**, **j**) and bloating (**k**, **l**) in younger (*n*=10) and older (*n* = 10) men immediately before and after drinks containing water (control) and 30 g whey protein. Time (0–180min) effects were determined by using mixed effects analysis. Ratings of fullness, hunger, nausea, and bloating did not change from baseline during all study days in both age groups (*P* > 0.05). Scores for prospective food consumption were significantly higher in the young on the control day (*P* = 0.040), whereas in the older group, scores were significantly higher on the protein day (protein effect *P* = 0.004*;* age x protein interaction effect *P* = 0.001). For desire to eat prospective consumption was significantly higher in the younger than older group on the control day (age effect *P* = 0.040), but not on the protein day (age effect *P* = 0.98; age×protein interaction effect *P* = 0.020).

The age × protein interaction effect for ratings of hunger, fullness, prospective food consumption, nausea, and bloating were not significant (*P* > 0.05), although there was a strong trend for desire to eat to be less in the older subjects (age × protein interaction effect *P* = 0.051; Fig. 4).

## Discussion

The major findings of this study are:

1. There was no suppression of *ad libitum* energy intake after 30 g whey protein ingestion in either older or young, obese men. This is in contrast to our previous studies in lean, non-obese men, where greater (and significant) suppression of energy intake was observed in younger than older men, after both a 30 g whey protein drink (1% vs. 15% suppression)^33^ and intraduodenal whey infusions (1% vs. 19% suppression)^17^.
2. The whey protein drink slowed gastric emptying, to a comparable degree in both age groups.
3. There was no effect of the protein drink on hunger, desire to eat, fullness, satiety and bloating.

Numerous studies, including our own, have shown that ingestion of protein, by mouth and directly into the stomach or small intestine, acutely suppresses appetite and ad libitum energy intake in young, non-obese adults. Evidence that protein is the most satiating of the macronutrients in young adults^27,28^, has led to the development of high protein (usually energy-restricted) diets and their recommendation to young, overweight people trying to lose weight. Increased protein intake in these diets is often at the expense of reduced carbohydrate intake^48–50^. Many people, however, fail to achieve substantial, long-term weight loss on such diets. One possible explanation for this is that protein is not as satiating in obese as in non-obese individuals. In the present study we examined the impact of obesity on the satiating effects of protein ingestion^33^. In our previous study of young, non-obese men (mean BMI 23 kg/m^2^) we showed that a 30 g whey protein drink significantly suppressed *ad libitum* energy intake at a test meal 3 h later by 17% compared to control day intake. That suppression was associated with reduced appetite ratings. In contrast, in the present study, using the same whey dose and study protocol, the protein drink suppressed neither energy intake nor appetite ratings in young *obese* men.

These results contrast with those of Brennan et al.^51^, who reported that high protein meals suppress energy intake in both lean and obese younger men 3 h after an energy preload. While the energy preloads in that study were larger than we employed (213 kcal vs. 120 kcal), and administered as a fat, protein and carbohydrate mixture, we have reported previously that pure whey drinks of 280 kcal and mixed macronutrient drink of 504 kcal protein (280 kcal), carbohydrate (112 kcal) and fat (112 kcal) drinks also did not suppress voluntary energy intake at 3 h in non-obese older men^16^. The preloads in Brennan et al. were solid food (a meat and pasta dish), compared to a whey drink in our study. There is evidence that drinks are less satiating than solid foods of the same nutrient composition^51^. Mourao et al., for example, reported greater *ad libitum* energy intake after drinks than solid food of comparable energy content^52^. These results are consistent with the possibility of a reduced satiating effect of liquid vs. solid protein. This would support the use of increased oral protein in solid rather than liquid form when the intention is to promote weight loss. Nevertheless, the whey protein drink *did* suppress appetite and food intake in young, non-obese men in our previous study, an effect not present in the obese young men in this study. These results suggest that obesity may blunt the satiating effects of protein, at least whey protein, and that these effects of obesity may be similar to those of physiological ageing; both healthy ageing and obesity have been associated with a loss of suppression of subsequent food intake by a whey drink.

The mechanism(s) by which obesity blunts the satiating effects of whey protein are not known. Blood glucose concentrations were predictably higher in the older than younger men, but not affected by protein ingestion, as in our previous study of non-obese men^33^, suggesting that glucose was not involved.

Gastric emptying was slowed by ingestion of the whey protein in both younger and older men with no difference between them. Despite this, subsequent food intake was suppressed by whey in neither age group. This suggests, at least under these study conditions, that gastric distension due to retention of the protein load in the stomach and delayed entry of test drink into the small intestine, did not affect appetite and food intake. To allow a comprehensive assessment of gastric emptying the test meal was started 180 min after the study drink, by which time the stomach was almost completely empty. It is possible that if the test meal was given earlier, when the difference between the study days in how much drink remained in the stomach was greater, there may have been reduced food intake on the whey drink day and an association between food intake and gastric emptying. Nevertheless, further evidence for the lack of such an association is provided by the results of our previous study in non-obese men^33^, in which gastric emptying was markedly slowed by the whey drink in both older and young men, with almost complete emptying of the drink from the stomach before buffet meal ingestion at 180 min. In that study, there *was* a reduction in ad libitum energy intake in the non-obese young men, suggesting that slowing of gastric emptying by protein ingestion is not involved in the satiating effect (or lack of) of the protein drinks under these study conditions.

Increasing age is associated with a physiological reduction in hunger and food intake, the so-called “anorexia of ageing”^41,53^. Consistent with that reduction, the older subjects consumed about 20% less energy in this study than the younger subjects, although that reduction was not statistically significant. On average, body weight decreases after about age 60–70 years. Much of the weight lost is lean tissue, including skeletal muscle and bone^54,55^. Muscle loss can be marked, particularly when pathological processes are superimposed, and can lead to sarcopaenia and frailty. One way to prevent this skeletal muscle loss, and thus preserve function and quality of life, is with dietary protein supplements, that can help maintain lean body mass and improve health^56^. In contrast, when body weight is deliberately lost with energy-reduced diets, lean tissue is lost as well as fat. This may have adverse effects in older people with baseline low muscle mass. The results of this and our previous acute studies suggest that protein supplementation as whey protein drinks can be given to older people as an aid to skeletal muscle preservation and even augmentation, without risk of appetite suppression and weight loss. Conversely, such protein drinks are likely to have little effect to promote weight loss in overweight, older people trying to lose weight, and are likely to be even less effective than in younger adults. Longer term studies in both obese and non-obese older adults evaluating the effects on appetite and food intake of increased protein intake in solid foods would be of interest.

Limitations of this study include the relatively modest subject numbers and the use of only one dose of whey protein. We did not assess the subjects’ perceptions of taste and/or pleasantness of the drinks. Gastric emptying rate was measured indirectly by 2D Ultrasound. 3D ultrasonography, which we have used previously^33,57,58^ is probably more accurate in non-obese people, but was not used here as the results are less reliable in obese individuals due to tissue layers. Scintigraphy is the ‘gold standard’ technique for measurement of gastric emptying but was not available for this study. We studied only men, as they appear to have the greatest ability to regulate energy intake in response to energy manipulation^34^. The results do not necessarily apply to women.

In summary a 30 g whey protein drink did not suppress appetite or energy intake in obese younger or older men. We speculate that obesity might mimic the effects of ageing to inhibit responses to protein. The use of liquid whey supplementation is unlikely to be helpful as a weight loss strategy in men of any age. Liquid whey protein supplements, the use of which may be of benefit for the preservation of function in older people, are unlikely to suppress food intake in older people of any weight.
